# Electroreduction of dissolved carbon dioxide on roughened molybdenum microelectrodes†

**DOI:** 10.1039/d3ra05592b

**Published:** 2023-11-08

**Authors:** Siti Hajjar Yahya, Firas A. Al-Lolage, Mohd Muzamir Mahat, Muhammad Zahir Ramli, Mohd Syamsul, Shaili Falina, Dania Adila Ahmad Ruzaidi, Wan Hazman Danial, Saiful Arifin Shafiee

**Affiliations:** a Department of Chemistry, Kulliyyah of Science, International Islamic University Malaysia Jalan Sultan Ahmad Shah, Bandar Indera Mahkota 25200 Kuantan Pahang Malaysia sabs@iium.edu.my; b Department of Chemistry, College of Science, University of Mosul Mosul 41002 Iraq; c Textile Research Group, Faculty of Applied Sciences, Universiti Teknologi MARA 40450 Shah Alam Selangor Malaysia; d Institute of Oceanography and Maritime Studies (INOCEM), Kulliyyah of Science, International Islamic University Malaysia Kampung Cherok Paloh 26060 Kuantan Pahang Malaysia; e Institute of Nano Optoelectronics Research and Technology (INOR), Universiti Sains Malaysia, Sains@USM Bayan Lepas 11900 Pulau Pinang Malaysia; f Collaborative Microelectronic Design Excellence Centre (CEDEC), Universiti Sains Malaysia, Sains@USM Bayan Lepas 11900 Pulau Pinang Malaysia; g IIUM Health, Safety, and Environment, Kulliyyah of Medicine, International Islamic University Malaysia Jalan Sultan Ahmad Shah, Bandar Indera Mahkota 25200 Kuantan Pahang Malaysia; h Sustainable Chemistry Research Group, Kulliyyah of Science, International Islamic University Malaysia, Jalan Sultan Ahmad Shah, Bandar Indera Mahkota 25200 Kuantan Pahang Malaysia; i School of Physics and Material Studies, Faculty of Applied Sciences, Universiti Teknologi MARA 40450 Shah Alam Selangor Malaysia

## Abstract

The increasing levels of carbon dioxide (CO_2_) in the atmosphere may dissolve into the ocean and affect the marine ecosystem. It is crucial to determine the level of dissolved CO_2_ in the ocean to enable suitable mitigation actions to be carried out. The conventional electrode materials are expensive and susceptible to chloride ion attack. Therefore, there is a need to find suitable alternative materials. This novel study investigates the electrochemical behaviour of dissolved CO_2_ on roughened molybdenum (Mo) microdisk electrodes, which were mechanically polished using silicon carbide paper. Pits and dents can be seen on the electrode surface as observed using scanning electron microscopy. X-ray diffraction spectra confirm the absence of abrasive materials and the presence of defects on the electrode surface. The electrochemical surface for the roughened electrodes is higher than that for the smoothened electrodes. Our findings show that the roughened electrodes exhibit a significantly higher electrocatalytic activity than the smoothened electrodes for the reduction of dissolved CO_2_. Our results reveal a linear relationship between the current and square root of scan rate. Furthermore, we demonstrate that saturating the electrolyte solution with CO_2_ using a bubbling time of just 20 minutes at a flow rate of 5 L min^−1^ for a 50 mL solution is sufficient. This study provides new insights into the electrochemical behaviour of dissolved CO_2_ on roughened Mo microdisk electrodes and highlights their potential as a promising material for CO_2_ reduction and other electrochemical applications. Ultimately, our work contributes to the ongoing efforts to mitigate the effects of climate change and move towards a sustainable future.

## Abstract 2

SiC roughened Mo microdisk electrodes show a higher electrocatalytic activity than smoothened Mo electrodes.

## Graphical abstract

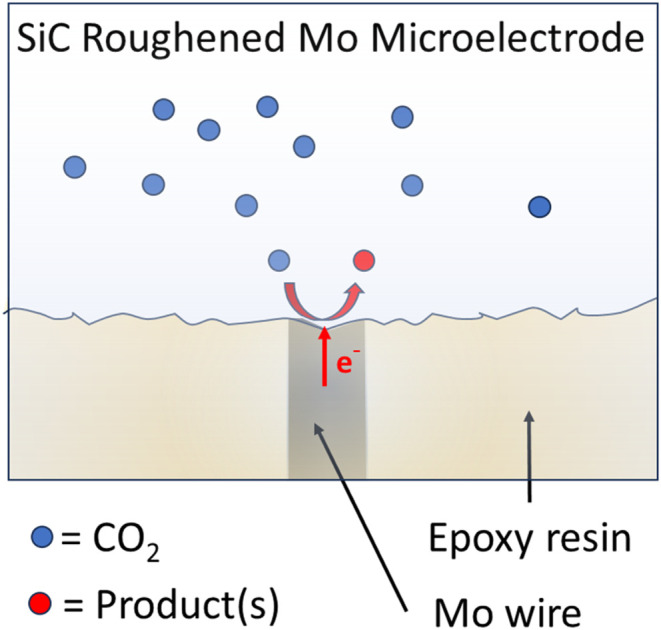

## 1 Introduction

Greenhouse gases (GHG) are the main cause of climate change and global warming that could disrupt the entire ecosystem. The main GHGs in the atmosphere emitted by human activities are water vapor, carbon dioxide (CO_2_), methane (CH_4_), nitrous oxide (N_2_O), and ozone (O_3_).^1^ However, CO_2_ has drawn attention from the global community because it is the most abundant and well-known greenhouse gas.^2^ Since 1950, the amount of CO_2_ in the atmosphere has significantly increased by approximately 30% due to anthropogenic activities.^3^ The average temperature has increased by around 1 °C since 1880, which correlates with the increase in atmospheric CO_2_ from 280 ppm which was recorded prior to the start of the industrial revolution to 410 ppm by 2013.^4^ Over the most recent ten years, the growth rate of CO_2_ was known to be around 2.3 ppm per year, which is about 100 times faster than that of CO_2_ coming from natural occurrence.^5^ Atmospheric CO_2_ may dissolve into the ocean and this phenomenon has resulted in several devastating consequences. The dissolved CO_2_ forms carbonic acid which then readily dissociates into bicarbonate (HCO_3_^−^), carbonate (CO_3_^2−^), and protons (H^+^); this lowers the pH value, changing it from its current alkaline pH which is around 8.1, making it more acidic.^6^ The concentration of H^+^ in the oceans has increased by approximately 30% in the last 150 years due to the high amount of dissolved CO_2_. Noor and Das^7^ have reviewed the negative effects of elevated dissolved CO_2_ on marine ecosystems. Many coral reefs have been significantly affected by this event as they are very sensitive to changes in pH and temperature.^8^ Coral reefs are an essential habitat for millions of marine species. Therefore, alterations in their structure could lead to an unbalanced ecosystem, which would also significantly impact the food chain.

As compared to chromatographic and spectrometric techniques such as Fourier transform infrared, electrochemical sensors are simple in their setup.^9^ Unfortunately, the current noble metal sensors for electrochemical detection such as gold are expensive and susceptible to damages due to the presence of chloride ions in the electrolyte. Meanwhile, the drawbacks of employing metal oxide materials as the electrode material are mostly their elevated operating temperature from 200 to 600 °C and cross-sensitivity towards carbon monoxide, oxygen, hydrogen, and even humidity.^10^ Other electrochemical techniques such as Severinghaus-type electrodes have pH drift problems which make the obtained results to be unreliable.^10^ Meanwhile, glass electrodes are difficult to miniaturise, brittle, and expensive which may pose safety risks due to the sharp glasses and may use a lot of spaces.^11^ Therefore, there is a need to find an alternative material to measure the concentration of dissolved CO_2_.

The electrochemical reduction of dissolved CO_2_ may proceed in different ways. Dissolved CO_2_ is electrochemically reduced to yield different products which involves different number of electrons transferred.^12^Table 1 summarises the possible electrochemical reactions for CO_2_. Therefore, many groups have reported the potential of electrocatalysts to convert CO_2_ into useful products such as formaldehyde, methanol, methane, carbon, formic acid, and more.^12^ This has been suggested as one of the ways to reduce the amount of CO_2_ in the environment which is hoped to reverse the effects of global warming and climate change.

**Table 1:** Reaction pathways for the electrochemical reduction of CO_2_ along with their respective standard potential^12^

Reaction pathway	Potential^a^ (*vs.* SHE/V)
CO_2_ + 4H^+^ + 4e^−^ → C + 2H_2_O	0.210
CO_2_ + 2H_2_O + 4e^−^ → C + 4OH^−^	−0.627
CO_2_ + 2H^+^ + 2e^−^ → HCOOH	−0.250
CO_2_ + H_2_O + 2e^−^ → HCOO^−^ + OH^−^	−1.028
CO_2_ + 2H^+^ + 2e^−^ → CO + H_2_O	−0.106
CO_2_ + H_2_O + 2e^−^ → CO + 2OH^−^	−0.934
CO_2_ + 4H^+^ + 4e^−^ → CH_2_O + H_2_O	−0.070
CO_2_ + 3H_2_O + 4e^−^ → CH_2_O + 4OH^−^	−0.898
CO_2_ + 6H^+^ + 6e^−^ → CH_3_OH + H_2_O	0.016
CO_2_ + 5H_2_O + 6e^−^ → CH_3_OH + 6OH^−^	−0.812
CO_2_ + 8H^+^ + 8e^−^ → CH_4_ + 2H_2_O	0.169
CO_2_ + 6H_2_O + 8e^−^ → CH_4_ + 8OH^−^	−0.659
2CO_2_ + 2H^+^ + 2e^−^ → H_2_C_2_O_4_	−0.500
2CO_2_ + 2e^−^ → C_2_O_4_^2−^	−0.590
2CO_2_ + 12H^+^ + 12e^−^ → CH_2_CH_2_ + 4H_2_O	0.064
2CO_2_ + 8H_2_O + 12e^−^ → CH_2_CH_2_ + 12OH^−^	−0.764
2CO_2_ + 12H^+^ + 12e^−^ → CH_3_CH_2_OH + 3H_2_O	0.084
2CO_2_ + 9H_2_O + 12e^−^ → CH_3_CH_2_OH + 12OH^−^	−0.744

a Equilibrium potential at 25 °C and 100 kPa, proton activity of 1 mol L^−1^. SHE = standard hydrogen electrode.

Herein, we report the electrochemical behaviour of CO_2_ reduction on roughened molybdenum (Mo) microdisk electrodes which were prepared *via* mechanical polishing. Therefore, the innovative part of the work presented herein is the preparation of roughened Mo microdisk electrodes using silicon carbide papers which are utilised to assess the electrochemical behaviour of dissolved carbon dioxide in aqueous solutions. To the best of our knowledge, there is no group that has studied the electrochemical behaviour of CO_2_ on this type of microelectrode. Roughening the microelectrode surface by mechanical polishing method can increase the amount of active sites to improve both sensitivity and response time without expensive surface modifications.^13^ This work introduces a low-cost and convenient method for the fabrication of roughened Mo microdisk electrodes. Microdisk electrodes are well-known for their unique characteristics such as low ohmic drop, high signal-to-noise ratio, fast response time, and high spatial resolution. Therefore, it would be interesting to assess the voltammetric features of dissolved CO_2_ on roughened Mo microdisk electrodes.

## 2 Materials & methods

### 2.1 Preparation of roughened Mo microelectrodes

Mo wires (Good Fellow, 99.95%) were cut into small pieces of about 2 to 3 cm in length. The wires were cleaned using acetone and deionized water. Subsequently, the wires were dried in ambient air. One end of each cleaned Mo wire was later connected to a multicore insulated copper (RS Components) using silver paint (XeredEx). The silver paint was then left until it is completely dry. An epoxy resin (Daewoo) was utilized to cover the wires and acted as an insulating sheath. In addition, one end of the hardened epoxy resin was affixed to a hollow borosilicate tube using more epoxy resin for easier handling. The prepared electrodes were then polished with abrasive materials until the wires were exposed. To obtain a smooth electrode surface, the Mo microelectrodes were polished with abrasive materials of different grades: 500, 800, 1200, 2000, 2500, 8000, and 25 000 grit. Abrasive waterproof sandpapers (Nippon paint & TAGA) and alumina lapping films (Allied High Tech) were used as the polishing materials. However, their initial grades have been converted into Japanese Industrial Standard (JIS) to avoid confusion. This standard was chosen because it covers a wide range of grades. As a precautionary step, the electrode surface was cleaned using a wetted polishing microcloth (Buehler) and rinsed with deionized water before changing to a lower grade of the polishing materials; this was done to remove any residues of the abrasive materials from the electrode surface. Meanwhile, roughened Mo microelectrodes were prepared by polishing the electrode surface with grade 500 grit silicon carbide paper for 1 minute. The electrodes were polished in a figure 8 motion whilst making sure that the electrodes were perpendicular to the surface to ensure that the electrode tips were flat on the surface of polishing materials. Care must be taken as the electroactive area could end up having a different electroactive dimension if the electrode was polished at an angle.

### 2.2 Physical characterization of Mo wires

The morphology of the microelectrodes polished with the 25 000 grit of abrasive material was compared to that of the electrodes roughened using the 500 grit of abrasive materials using an optical microscope (Olympus) and a scanning electron microscope (SEM, Zeiss EVO MA15). To this aim, the wires were sealed in epoxy resins inside a mould. The hardened epoxy resins were not attached to hollow borosilicate tubes to easily fit inside the chamber. The hardened epoxy resin underwent the same aforementioned processes to obtain smoothened and roughened Mo wires. Because the epoxy resin was utilised as the insulating sheath, most of the electrode tip was not conductive which may lead to the charging of the surface. Therefore, the microelectrode surface was sputtered with a thin layer of gold to reduce the charging effect. Each sputtered epoxy resin was placed on a carbon tab with the wires facing upward and the tab was affixed to a pin stub. The pin stub was put on a platform inside the SEM chamber and SEM images were taken at different magnifications.

To confirm that there was no contamination coming from the abrasive materials, an elemental analysis was conducted on virgin, smoothen, and roughened wires using an X-ray diffractometer (XRD, PANalytical X-Pert Pro). XRD spectra were collected from the wires that were sealed inside the epoxy resins without the hollow borosilicate tubes for both smoothened and roughened wire. As for the virgin sample, the XRD pattern was recorded on the commercially available wire in its original state. This study was performed to make sure that the reduction of CO_2_ was not influenced by the presence of contaminants on the surface of the exposed electroactive materials especially from the abrasive materials.

### 2.3 Electrochemical characterization

The electrochemical experiments were conducted using a 910 PSTAT Mini potentiostat (Metrohm) inside a grounded Faraday cage (Metrohm). All chemicals were used as received without any further purification. Saturated calomel electrodes (SCE) and platinum (Pt) sheets were used as the reference and counter electrodes respectively. To assess the electrochemical behaviours of dissolved CO_2_ on the roughened and smoothened surface microelectrodes, a solution of 0.5 M KCl was first purged with purified nitrogen (N_2_) gas (Maxdeal) for 20 minutes to remove any dissolved gases. Cyclic voltammetry (CV) was performed by cycling the potential from 0.1 V until −1.2 V *vs.* SCE at 10 mV s^−1^. Subsequently, CO_2_ gas was passed through the solution for 20 minutes before running the next electrochemical measurements.

The electrochemical behaviours of CO_2_ reduction were assessed on smooth and roughened Mo microdisk electrodes in 0.5 M KCl at 10 mV s^−1^. A scan rate study was also conducted using the roughened Mo microdisk electrodes where CVs were recorded in 0.5 M KCl at different scan rates: 10, 20, 50, 100, 200, 500, and 1000 mV s^−1^. Other than that, voltammetric responses were also obtained after bubbling the electrolyte solutions with CO_2_ gas for different periods of time.

## 3 Results and discussions

### 3.1 Optical microscopy

**Fig. 1:**
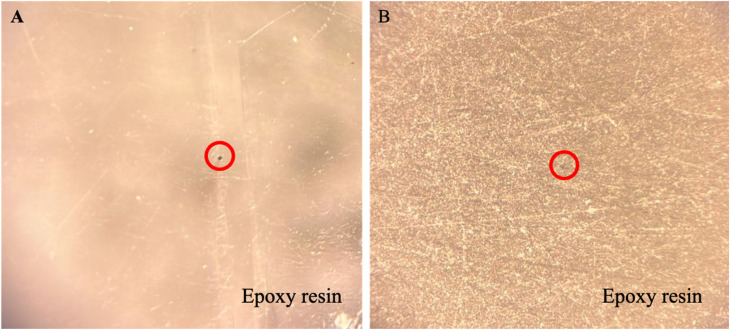
The optical microscope images of the (A) smoothened and (B) roughened electrode tip taken at 100× magnification. The red circle refers to the wire in the epoxy resin.

The Mo wires were turned into microelectrodes and polished with different grades of abrasive materials to have a different surface roughness. Then, they were characterised using optical microscopy. Fig. 1 shows the morphology of Mo microelectrodes that have been polished with polishing materials of 500 grit and 25 000 grit. Unfortunately, the highest magnification that can be set for the optical microscope was only up to 100×. The red circles in Fig. 1 pointed to the location of the Mo wires.

Fig. 1(A) shows a smooth surface of the electrode tip. There are a few fine lines which could be coming from foreign matters with large dimensions present on the electrode surface or the alumina lapping films during polishing. There are also a few dents in the epoxy resin which could be coming from small bubbles formed during mixing. However, these features are acceptable as they will not affect the electroactive area or the electrochemical process. As expected, the electrode tip that was polished with the roughest abrasive material exhibits many scratches with a few dents as shown in Fig. 1(B). This is to show the effect of using polishing materials with different grades on the epoxy resin. The next section discusses the morphology of the electroactive area.

### 3.2 Scanning electron microscopy (SEM)

**Fig. 2:**
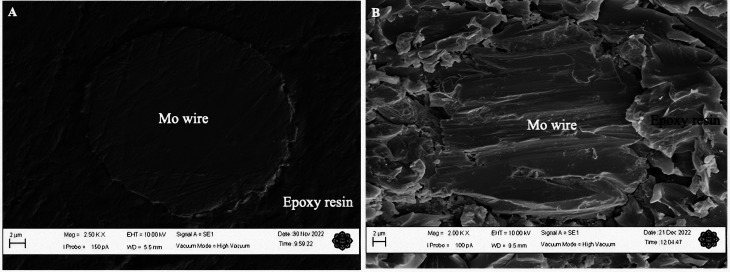
The SEM images of a Mo electrode with a smooth and rough surface after polishing it with an abrasive material of 25 000 and 500 grit respectively. Epoxy resin was used as the insulating sheath for the Mo wire which is also shown in the SEM images. Both images were taken using a secondary electron detector and high vacuum mode. Different conditions that were employed to obtain the images: (A) magnification = 2500×; working distance = 5.5 mm and (B) magnification = 2000×; working distance = 9.5 mm. The scale bars depicted in the figures correspond to a length of 2 microns.

This study was done to analyse the morphology of the microelectrode surface at high magnifications. The electrodes were thoroughly cleaned with a wetted microcloth and rinsed with deionised water after polishing them. Fig. 2 shows the SEM micrographs of the Mo microelectrodes with a smooth surface taken at 2500× and a roughened surface obtained at 2000×.

**Fig. 3:**
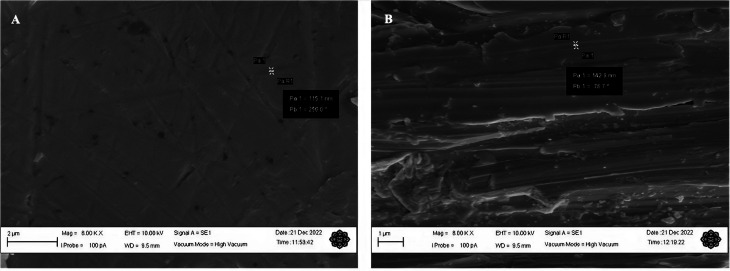
The SEM images of the smoothened and roughened Mo wires that were polished using abrasive materials of (A) 25 000 and (B) 500 grit. The images were taken at 8000× magnification. The images were obtained using a secondary electron detector under high vacuum mode. The applied working distance for both samples was 9.5 mm. The scale bars shown indicate the dimensions of (A) 2 μm and (B) 1 μm.

Some fine scratch lines and bumps can be seen clearly on the smooth electrode surface as shown in Fig. 2(A) which implies that it was thoroughly polished. However, Fig. 2(B) demonstrates a very rough surface with a few defects on the surface of Mo wire. Meanwhile, the hardened epoxy resin has a rougher surface than the Mo wire with crumble- and flake-like structures. Nonetheless, this process did not expose the side of the wire which could increase the electroactive area. SEM images for both smoothened and roughened Mo wires taken at 8000× are provided in Fig. 3.

Small scratches can clearly be seen on the smoothened Mo wire in Fig. 3(A) as previously observed in Fig. 2(A). In addition, some surface defects such as small pits and dents can also be observed in the figure. These surface features are typical even after polishing with the lowest grade of polishing materials.^14,15^ The scratch marks for roughened Mo microelectrodes can be seen clearly on the surface of the wire as shown in the SEM image in Fig. 2(B). On average, the scratch lines on the smoothened and roughened Mo wires have the width of around 90 and 140 nm respectively. Fig. 2(B) also shows that the surface of Mo wire was also uneven with many small steps and dents unlike the smoothened microelectrode. This feature may provide more active sites on the electroactive surface.

### 3.3 X-ray diffraction (XRD)

**Fig. 4:**
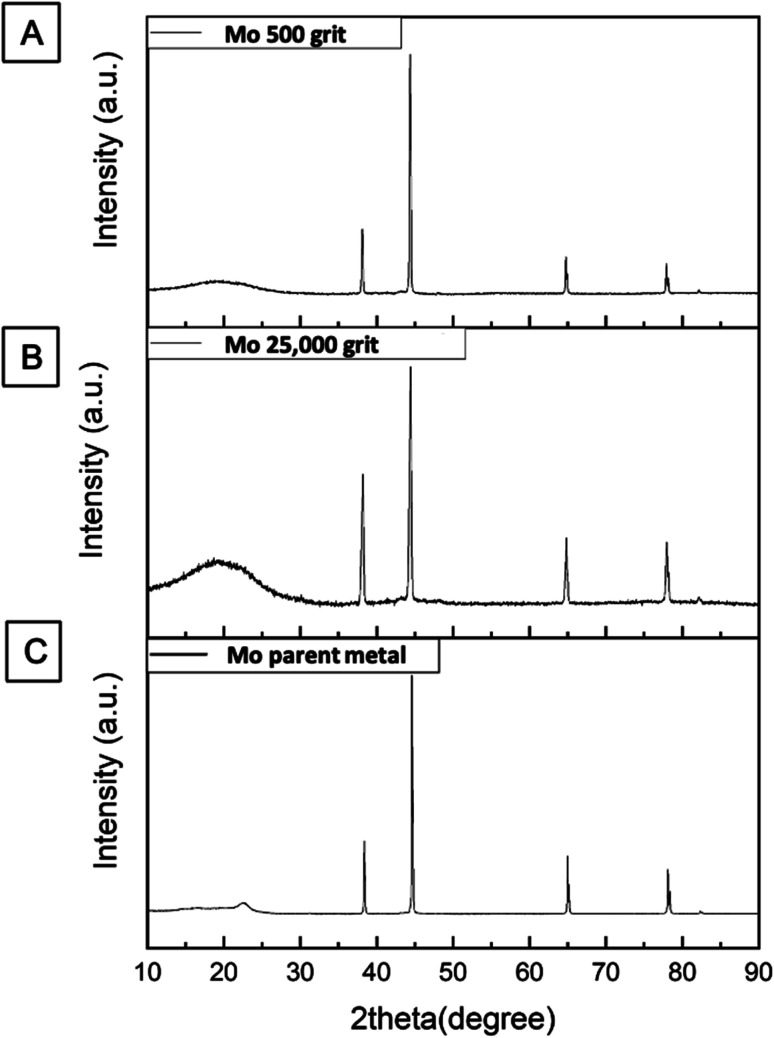
The XRD patterns for Mo microdisk electrodes that were polished with (A) 500 and (B) 25 000 grits polishing materials including one for a (C) Mo parent metal without polishing.

X-ray diffraction (XRD) was performed on both types of electrodes to determine whether the electroactive surface has been contaminated with the polishing materials. XRD spectra obtained on the smoothened and roughened Mo wires are shown in Fig. 4. The electrodes were polished as described in Section 2.1. The electrodes were properly cleaned by rinsing them with deionised water and a wetted polishing cloth before placing the samples inside the XRD chamber.

The XRD spectrum recorded for the roughened Mo wire is similar to that of smoothened Mo electrodes in terms of the locations of the peaks. When compared to the XRD spectrum of Mo wire without any modifications, the pattern is also fairly similar to both spectra shown in Fig. 4. This suggests that the polishing material can be thoroughly cleaned from the electrode surface and they do not change the composition of the electrode materials during polishing. Both silicon carbide papers and alumina lapping films only change the morphology of the electrode surface. Therefore, the electroreduction of dissolved CO_2_ discussed in the next section can be said to be solely coming from the interaction between the electroactive material and dissolved CO_2_ molecules.

### 3.4 Electroreduction of dissolved carbon dioxide

**Fig. 5:**
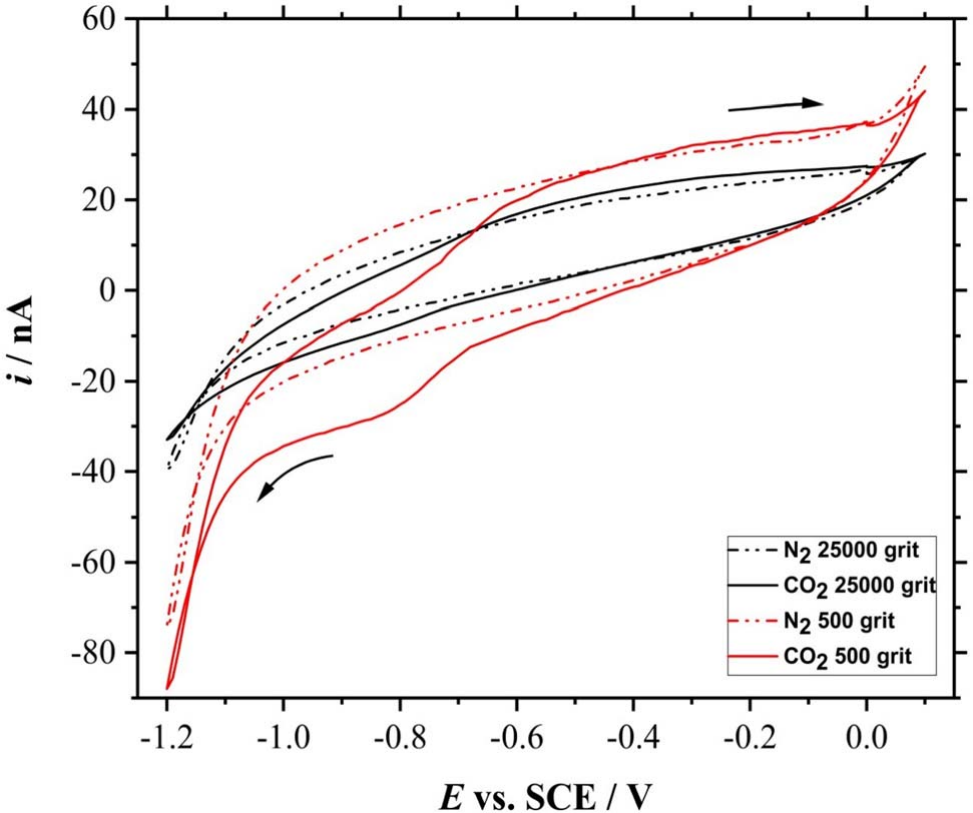
CVs (3^rd^ cycle) acquired with the 25 μm ∅ Mo microdisk electrodes in N_2_-saturated (dash-dot lines, -·-) and CO_2_-saturated (solid lines, -) 0.5 M KCl solutions at 0.01 V s^−1^. The electrodes were polished with polishing materials of 500 (red) and 25 000 (black) grits respectively.

The electroreduction of CO_2_ was assessed on the smoothened and roughened Mo microdisk electrodes. The electrodes were polished and cleaned accordingly as explained in the previous sections. CV was performed on both smoothened and roughened Mo microdisk electrodes in the presence and absence of dissolved CO_2_ where the results are shown in Fig. 5.

There is a difference between the CVs obtained in the presence and absence of dissolved CO_2_ for both smoothened and roughened Mo microdisk electrodes. This implies that the reduction peaks are coming from the electroreduction of dissolved CO_2_. However, the voltammogram recorded using the roughened electrode surface seems to show a higher electrocatalytic activity than the smoothened electrode surface. The foot of the reduction wave for the smoothened electrode surface is at around −0.6 V *vs.* SCE whereby the one for the roughened surface is at approximately −0.3 V *vs.* SCE. This could be due to a higher amount of active sites on the roughened electrode surface. It can also be observed that there are two reduction waves for the voltammetric response obtained using the electrode polished with the 500 grit abrasive materials where the first peak is at approximately −0.65 V *vs.* SCE and the second peak is at around −0.9 V *vs.* SCE. Most of the reduction peaks reported for the reduction of dissolved CO_2_ in aqueous solutions are beyond −1 V *vs.* SCE.^16^ For example, the reduction wave for the dissolved CO_2_ in a 0.2 M Na_2_SO_4_ solution on phosphorus-doped polycrystalline diamond electrodes was at approximately −1.64 V *vs.* SCE as reported by Naragino and colleagues.^17^ This suggests that the roughened Mo microdisk electrodes may have a higher electrocatalytic activity than the phosphorus-doped polycrystalline diamond electrodes for the reduction of dissolved CO_2_ in aqueous solutions. The electrochemical CO_2_ reduction does not require a high driving force when using roughened Mo microdisk electrodes as compared to the phosphorus-doped polycrystalline diamond electrodes. Another notable feature is that the limiting current for the dissolved CO_2_ obtained on the roughened Mo microdisk electrodes is around −18 nA at −0.9 V *vs.* SCE after subtracting it with the background current which is still larger than that recorded with a smoothened microdisk electrode which is around −3 nA at −0.9 V *vs.* SCE.

The concentration of any analytes can be approximated by the following mathematical equation which is specifically used for microelectrodes: *i*_lim_ = 4*nFDCa* where *i*_lim_ is the limiting current (Ampere), *n* is the number of electrons transferred, *F* is the Faraday constant (96 485 C mol^−1^), *D* is the diffusion coefficient of the analyte of interest (cm^2^ s^−1^), *C* is the concentration of the analyte (mol cm^−3^), and *a* is the radius of the electrode (cm).^18^ The diffusion coefficient of CO_2_ can be affected by several factors which include the concentration of test solution, temperature, and pressure.^19^ The diffusion coefficient of CO_2_ in 0.5 M KCl is approximately 1.76 × 10^−5^ cm^2^ s^−1^.^20^ Assuming that the number of electrons transferred is 2, the concentration of dissolved CO_2_ in the solution is 0.00424 M. As described in the introduction section, the electroreduction of dissolved CO_2_ can proceed *via* different pathways. The work presented herein is mainly to report on the electrochemical behaviour of dissolved CO_2_ on roughened Mo microdisk electrodes. Therefore, the product(s) of the electrochemical processes were not determined *via* any techniques such as chromatography so as to confirm the pathways taken by the reduction of dissolved CO_2_ on the roughened Mo microdisk electrodes.

The harsh treatment to roughen the electrode surface could not have exposed the side of Mo wire that could increase the electrode surface and lead to a larger peak current as evident from the SEM image shown in Fig. 2(B). This implies that the higher peak current for the roughened microdisk electrode could be more likely to be caused by an increase in the amount of active sites on the electrode surface as there are many defects such as steps and dents introduced on the surface as seen in Fig. 2(B) and 3(B). Although the position of the peaks in the XRD spectrum for the roughened Mo microdisk electrodes shown in Fig. 4(A) is similar to that for the pristine Mo wire which means that the crystal lattice did not change even after the harsh treatment, there was a significant difference in the intensity of the XRD signal between the Mo microelectrode polished with the 500 grit of abrasive material and the pristine wire which is also akin to the study reported by Zhang and colleagues.^21^ They reported that the samples with surface and subsurface defects have a lower signal intensity than the samples without defects when analysed using XRD. In our case, the signal intensity for the roughened Mo microdisk electrodes is lower than that for the pristine Mo wire which is an indicator for the presence of defects on the surface of the roughened electrodes. Therefore, the presence of defects such as pits and dents on the electrode surface of the roughened electrodes, which lowered the XRD peak intensity, could increase the number of active sites for the electroreduction of dissolved CO_2_. The electrochemical surface area (ECSA) was also assessed for the roughened and smoothened Mo microdisk electrodes by following the method that has been described by Trasatti and Petrii.^22^ The double layer capacitance calculated for the roughened and smoothened Mo microdisk electrodes were 514.79 and 249.65 nF respectively which were determined from the plots of current as a function of scan rate as depicted in Fig. S1.† Meanwhile, the ECSA of the electrodes which were determined from the double layer capacitance values for the roughened and smoothened Mo microelectrodes were 0.00858 and 0.00416 cm^2^ respectively. These values imply that the roughened Mo microelectrodes have a higher ECSA than the smoothened Mo microelectrodes which could be due to the defects introduced on the former electrodes.

The electrode tip was also polished with 500 grit for 3 minutes, but the current measured at −0.9 V *vs.* SCE is still around −18 nA (data not shown herein). This could mean that the surface of the electrode may be similar even after polishing the electrode with silicon carbide paper for a longer time. It is sufficient to just polish the electrodes for 1 minute to roughen the electrodes. Therefore, the electrodes were polished only for 1 minute. This finding is fairly beneficial due to the fact that the rough surface of silicon carbide paper could gradually reduce the length of electrode tip and wires during polishing.

### 3.5 Scan rate study

**Fig. 6:**
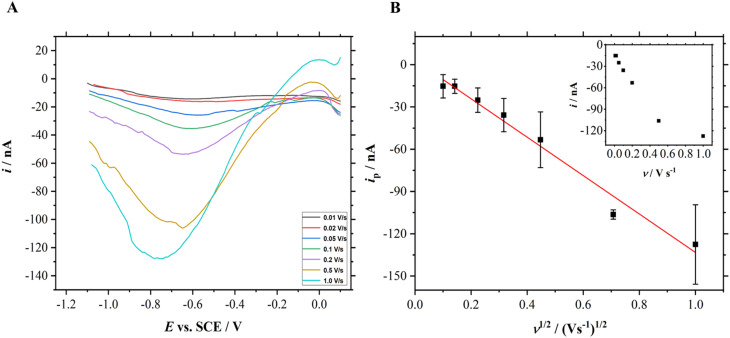
(A) Linear sweep voltammograms recorded in CO_2_ saturated 0.5 M KCl at different scan rates from 0.01, 0.02, 0.05, 0.1, 0.2, 0.5, and 1 V s^−1^. The voltammograms have been subtracted with their respective background currents. (B) A graph of peak currents for the reduction of dissolved CO_2_ on the roughened Mo microdisk electrode as a function of square root of scan rates. The inset is the plot of peak currents *vs.* scan rate. The red line denotes the linear fitting and the black scale bar at each data point represents the error bar.

Randles–Sevcik equation explains that the current should be directly proportional to the square root of scan rates for a reversible electrochemical process. Therefore, a scan rate study was performed using the roughened Mo microelectrodes in the presence of dissolved CO_2_ in the solution which is shown in Fig. 6. Linear sweep voltammograms were recorded using a roughened Mo microdisk electrode in N_2_- and CO_2_-saturated 0.5 M KCl solutions. The linear sweep voltammograms obtained in the presence of CO_2_ were then subtracted to those in the absence of CO_2_.

It can be seen from Fig. 6(A) that the magnitude of the current increases as the scan rate increases. At slow sweep rates, plateau regions could be seen on the voltammograms as demonstrated in Fig. 5 which corresponds to the hemispherical diffusion on microelectrodes. On the contrary, the reduction peaks recorded at fast scan rates do not exhibit the characteristic shape of voltammograms for microdisk electrodes even though the peak currents increase systematically. In principle, this could be attributed to planar diffusion control. A linear relationship between the current and the square root of scan rates as shown in Fig. 6(B) suggests that the reduction of dissolved CO_2_ on roughened Mo microdisk electrodes may not involve surface adsorption processes. The correlation coefficient that was obtained from the linear fitting is 0.993. This analysis is further corroborated by the plot of the peak currents *vs.* scan rate where the peak currents are not proportional to the scan rate as shown in the inset of Fig. 6(B).

### 3.6 Carbon dioxide gas bubbling time study

**Fig. 7:**
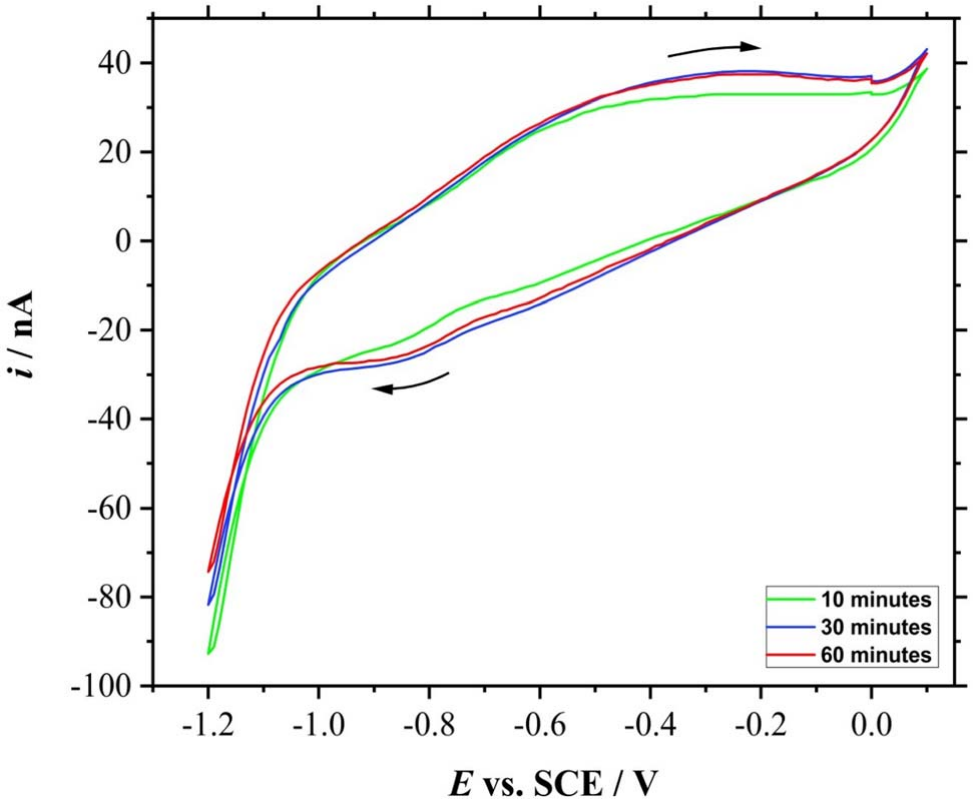
CVs (3^rd^ cycle) of a roughened 25 μm ∅ Mo microelectrode in 0.5 M KCl for 0.01 V s^−1^ with different CO_2_ bubbling time: 10 (green), 30 (blue), and 60 min (red).

CV was then performed after bubbling the 50 mL of electrolyte with CO_2_ gas at 5 L min^−1^ for a different period of time; 10, 30, and 60 minutes. In order to avoid the presence of other dissolved gases, N_2_ gas was bubbled for 20 minutes in between the experiments. Fig. 7 shows the voltammetric responses of the electroreduction of dissolved CO_2_ of different gas bubbling time.

The reduction peak for the 10 minutes of bubbling time is less than those for above 30 minutes of bubbling time. In fact, the CV for the roughened Mo microdisk electrode shown in Section 3.4 which was recorded after gassing the solution for 20 minutes also had a fairly similar peak currents to those CVs recorded after bubbling the test solution with CO_2_ gas for more than 30 minutes; their peak currents are approximately −18 nA taken at −0.9 V *vs.* SCE. The similarity in the peak currents suggests that 20 minutes was adequate to saturate the solution under the stated conditions. This is assuming that the gas flow rate remains the same throughout the experiment as a manual gas flow meter was used. Other than that, it was also assumed that other gases were not able to seep into the solution throughout the experiment as N_2_ gas was gently flowed to cover the surface of the solution without introducing disturbance. Moreover, the solution should contain no other gases as the solution was purged with N_2_ gas prior to the introduction of CO_2_ gas. As purified chemicals were used and each glassware was cleaned thoroughly, no contaminant should be present which could affect the test solution concentration and gas dissolution. The experiment was also performed at room temperature and pressure. Nonetheless, the time needed to saturate the solution with the analyte gas would also depend on the type and concentration of the electrolyte and the flow rate of the gas. This study is important to show the electrochemical response for different concentrations of CO_2_ which could open more research opportunities on enhancing the electrochemical performance of the electrode material.

## 4 Conclusions and potential future work

Roughened Mo microdisk electrodes were successfully prepared by polishing the electrode surface with a silicon carbide paper of 500 grit. The XRD patterns did not show any contamination of the electrode surface from the abrasive materials. They also exhibited the presence of microdefects on the surface of the electrodes after the harsh treatment which could provide more active sites for the electrochemical reduction of dissolved CO_2_ to occur. Interestingly, the roughened electrodes seemed to have a higher electrocatalytic activity than the smoothened electrode surface for the reduction of dissolved CO_2_ as the roughened microelectrodes needed a lower driving force than the smoothened electrodes for the reaction to proceed. The scan rate study revealed that the electroreduction of dissolved CO_2_ may not involve adsorbed species based on the linear relationship between current and square root of scan rates. For a solution of 50 mL, 20 minutes of CO_2_ bubbling time at 5 L min^−1^ seemed to be enough to saturate the solution because the cyclic voltammograms started to be reproducible. This electrode could be a potential candidate for other electrochemical reactions. However, more studies need to be done to further understand the electrochemical behaviour of many possible analytes on this type of electrode. The work could be extended to assess the electrochemical behaviour of dissolved CO_2_ using roughened Mo microdisk electrodes with different radii. The limiting current should be proportional to the radius of microdisk electrodes. This study will provide more insight on the reversibility of the electrochemical process. Another possible study is to assess the electrochemical behaviour of dissolved CO_2_ using roughened copper microdisk electrodes as copper electrodes are known to exhibit a high electrocatalytic activity for this reaction.^23–25^ The harsh treatment recommended in this work could enhance the electrochemical responses obtained on conventional copper electrodes due to the presence of defects on the electrode surface. As aforementioned, the presence of defects on the electrode surface may increase the number of active sites which is needed for the dissolved CO_2_ redox processes.

## Author contributions

Siti Hajjar Yahya and Dania Adila Ahmad Ruzaidi did the data curation, formal analysis, and investigation work. Saiful Arifin Shafiee and Firas A. Al-Lolage worked on the funding acquisition, project administration, supervision, resources, and writing – review & editing. Meanwhile, Muhammad Zahir Ramli, Mohd Muzamir Mahat, Mohd Syamsul, Shaili Falina, and Wan Hazman Danial did the writing – review & editing.

## Conflicts of interest

There are no conflicts of interest to declare.

## Supplementary Material

RA-013-D3RA05592B-s001
