# Supplementary material for: Electroreduction of dissolved carbon dioxide on roughened molybdenum microelectrodes
Source: RSC Adv. 2023 Nov 8;13(47):32918–26. doi: 10.1039/d3ra05592b (PMC10630746; doi:10.1039/d3ra05592b)
Supplement: RA-013-D3RA05592B-s001 [file RA-013-D3RA05592B-s001.pdf]

Electronic Supplementary Material (ESI) for RSC Advances.
This journal is © The Royal Society of Chemistry 2023

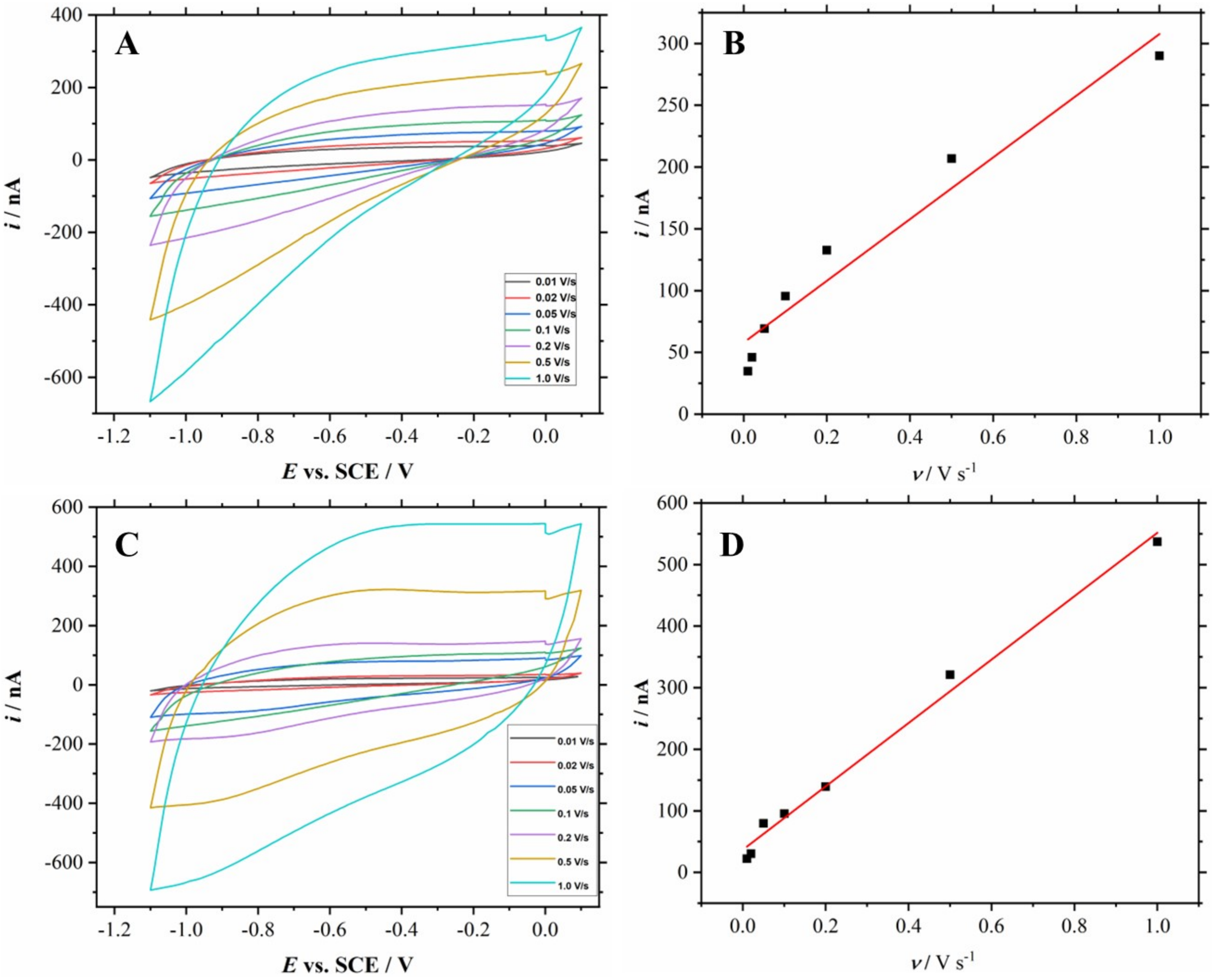

Figure S1: Cyclic voltammograms of smoothened (A) and roughened (C) Mo microdisk electrodes in N_2-saturated 0.5 M KCl at different scan rates: 0.01, 0.02, 0.05, 0.1, 0.2, 0.5, and 1 V s^{-1}. Corresponding plots of current as a function of scan rate for the smoothened (B) and roughened (D) Mo microdisk electrodes. The currents were taken at around -0.2 V *vs*. SCE and the red lines resemble the fitting of the data.
